# Integrating technical and political views for a sustainable European Distributed Infrastructure on Population Health

**DOI:** 10.1186/s13690-022-00790-w

**Published:** 2022-01-17

**Authors:** Alicia Padron-Monedero, Rodrigo Sarmiento Suárez, Petronille Bogaert, Linda Abboud, Herman Van Oyen, Hanna Tolonen, Mariken J. Tijhuis, Luigi Palmieri, Romana Haneef, Anne Gallay, Luis Lapao, Paulo Jorge Nogueira, Thomas Ziese, Stefanie Seeling, Jakov Vukovic, Isabel Noguer-Zambrano

**Affiliations:** 1 National School of Public Health, Instituto de Salud Carlos III. Av/ Monforte de Lemos 5, 28029 Madrid, Spain; 2 Department of Epidemiology and Public Health, Scientific Institute of Public Health. Sciensano, Brussels, Belgium; 3 Department of Public Health and Primary Care, Faculty of Medicine, Ghent University, Ghent, Belgium; 4 Department of Public Health and Welfare, Finnish Institute for Health and Welfare (THL), Mannerheimintie 166, 00271 Helsinki, Finland; 5 National Institute for Public Health and the Environment (RIVM), Bilthoven, Netherlands; 6 Department of Cardiovascular, Endocrine-metabolic Diseases and Aging, Istituto Superiore di Sanità (ISS), Via Giano della Bella, 34, 00161 Rome, Italy; 7 Santé Publique France, Saint-Maurice, France; 8 Institute of Hygiene and Tropical Medicine, Universidade NOVA de Lisboa, Lisbon, Portugal; 9 Instituto de Medicina Preventiva e Saúde Pública, Faculdade de Medicina da Universidade de Lisboa, 1649-028 Lisbon, Portugal; 10 Robert Koch Institute, Berlin, Germany; 11 Croatian Institute of Public Health (CIPH), Zagreb, Croatia

**Keywords:** InfAct, Health information, Health systems performance, Non- communicable diseases, Distributed infrastructure on population health

## Abstract

**Background:**

Non-Communicable diseases (NCD) are the main contributors to mortality and burden of disease. There is no infrastructure in Europe that could provide health information (HI) on Public Health monitoring and Health Systems Performance (HSP) for research and evidence-informed decision-making. Moreover, there was no EU and European Economic Area Member States (EU/EEA MSs) general consensus, on developing this initiative and guarantee its sustainability. The aim of this study is to analyze the integration of technical and political views made by the Joint Action on Health Information (InfAct; Information for Action) and the results obtained from those activities, in terms of advice and national and institutional support to develop an integrated and sustainable European Distributed Infrastructure on Population Health (DIPoH) for research and evidence-informed policy-making.

**Methods:**

InfAct established two main boards, the Technical Dialogues (TDs) and the Assembly of Members (AoM), to provide a platform for discussion with EU/EEA MSs to establish a sustainable infrastructure for HI: 1) The TDs were composed by national technical experts (NTE) with the aim to discuss and provide feedback about scientific aspects, feasibility and EU-added value of the infrastructure proposed by InfAct. 2) The AoM gathered country representatives from Ministries of Health and Research at the highest political level, with the aim of providing policy-oriented advice for the future political acceptance, support, implementation, and development of InfAct’s outcomes including DIPoH.

The documentation provided for the meetings consisted in Fact-Sheets, where the main results, new methods and proposals were clearly exposed for discussion and assessment; altogether with more extended information of the DIPoH. The documentation was provided to national representatives within one more before each TD and AoM meeting.

The Agenda and methodological approaches for each TD and AoM meeting consisted in the presentations of the InfAct outcomes extending the information provided in the Fact-Sheets; followed by a non-structured interaction, exchange of information, discussion and suggestions by the MSs representatives.

The outcomes of the non-structured discussions were collected in Minutes of the TD and AoM meetings, and the final version was obtained with the consensus of all participants. Additionally, structured letters of political support were provided to the AoM representatives, for them to consider providing their MS written support for DIPoH.

**Results:**

NTE, within the TDs, considered that DIPoH was useful for technical mutual learning and cooperation among and within countries; although they considered that the technical feasibility to uptake InfAct deliverables at the national and EU level was complex. The AoM focused on political support, resources, and expected MSs returns. The AoM representatives agreed in the interest of setting up an integrated and sustainable HI infrastructure and they considered DIPoH to be well-articulated and defined; although, some of them, expressed some barriers for providing DIPoH political support. The AoM representatives stated that the AoM is the most suitable way to inform EU MSs/ACs about future advances of DIPoH. Both boards provided valuable feedback to develop this infrastructure. Eleven countries and sixteen institutions supported the proposal, either by letters of political support or by signing the Memorandum of Understandings (MoU) and three countries, additionally, provided expression of financial commitment, for DIPoH to be added to the ESFRI 2021 roadmap.

**Conclusions:**

TDs and AoM were key forums to develop, advise, advocate and provide support for a sustainable European research infrastructure for Population Health.

## Background

The Council of the European Union (EU) [1] recommended in 2013 to the Commission of the EU and to the Member States (MSs) to establish an integrated and sustainable EU Health information system (HIS) focussed on Population Health and Health Systems Performance (HSP). However, this recommendation was not included in the agenda of main priorities of the MSs. Moreover, there was no general consensus at the EU and European Economic Area Member States (EU/EEA MSs) on how to develop this initiative and guarantee its sustainability. Although, some level of agreement had been previously outlined [2–4] (the health information system infrastructure should facilitate interaction between networks and experts, it should be distributed although the coordination could be performed by a central hub, and the infrastructure should provide access to high quality interoperable data for population health research and evidence based policies) [2–4].

A previous initiative to establish a shortlist of public health indicators serving as the core of a European public health monitoring system started in 1998 in response to a European Commission (EC) ‘s call. Several EU-funded projects developed the list of European Core Health Indicators (ECHI), and the first version of the ECHI shortlist was approved by the EC and the EU MSs in 2005. In 2008, a project funded by the Second Health Programme of the EC (2008–2013) stated that the EC and the EU MSs could put the indicators into practice with the aim of monitoring and comparing a limited number of health outcomes among EU countries, and to support evidence-informed policy-making [5]. However, this valuable initiative lacked sustainability at the EU level, due to an absence of updating and a definite governance structure beyond its funding period.

Another project, funded by the Third Health Programme of the EC (2014–2020), “Bridging Information and Data Generation for Evidence-based Health policy and research” (BRIDGE Health), was carried out from 2015 to 2017 with the aim to assess different structural and institutional options to develop a sustainable and integrated EU-HIS for both public health and research purposes. The BRIDGE Health project successfully developed the first proposal for the core elements (scope, tasks, activities, and governance) for an EU-HI research infrastructure [2–4].

Building on the Council of the EU recommendation [1] and the experience and results from the aforementioned initiatives [2, 5], the Joint Action on Health Information (InfAct, Information for Action) [6] was launched in March 2018 to run for three years (2018–2021). InfAct brings together 40 institutions from 28 EU/EEA MSs with the aim of developing an integrated and sustainable EU-HI infrastructure supporting country knowledge and capacities for health research and evidence-informed policy-making. To involve political and Technical Experts from EU/EEA MSs was considered necessary, in order to decide collaboratively whether an integrated and sustainable European Distributed Infrastructure on Population Health (DIPoH) was the best option for a EU-HI infrastructure among the several options investigated [2], and to gather their political and technical advice, acceptance and long-term support.

This paper aims to analyze the activities within InfAct to integrate technical and political views and the results obtained from those activities, in terms of advice and support, from EU/EEA MSs for innovate HI and setting up DIPoH, as a way forward to ensure sustainability beyond EU funded projects.

## Methods

InfAct has established two main boards: Technical Dialogues (TDs) and the Assembly of Members (AoM). The main objectives of these boards were to ensure engagement with EU/EEA MSs, to discuss, advice and generate consensus and support for a future sustainable EU-HI infrastructure. Both boards were established as follows:

### The Technical Dialogues (TDs)

TDs were composed by National Technical Experts (NTE), as representatives from EU/EEA MSs, and nominated from Ministry of Health (MoH) and Ministry of Research (MoR) authorities. InfAct’s recommendations for selecting the NTE were flexible to adapt to each MS singularity. InfAct’s recommendations included, as a general concept, to be in charge of their national HIS or working directly with them. A total of 15 EU/EEA MSs NTE representatives participated actively in the meetings. The participating countries were: Austria, Belgium, Croatia, Estonia, Finland, France, Germany, Italy, Ireland, Malta, Netherlands, Norway, Portugal, Spain, and Serbia.

The specific objectives of the TDs were: to provide technical feedback on activities of the InfAct project team, to assess the added value and feasibility to make DIPoH operational, and to raise awareness among decision makers.

Two TD meetings were held: October 2019 (Madrid) and September 2020 (virtual conference due to COVID-19 travel restrictions).

The InfAct outcomes are the products of each Work Package of the InfAct project team, in relation to DIPoH four core activities: “1. provide a one-stop-shop for population health data, 2. develop innovative methods, 3. build capacity, and 4. develop knowledge translation research” [7]. Each InfAct outcome was described and summarized, within a structured format, by the InfAct’s team responsible, in the form of Fact Sheets. In those Fact-Sheets, the main results, including proposals, guidelines, tools, and methods were clearly exposed for discussion and assessment (Table 1) [8, 9]. The Fact-Sheets were provided to the NTE within one more before each TD. Additionally, more extended information of the proposed new HI research infrastructure called DIPoH was also distributed [7]. DIPoH is designed as a HI distributed infrastructure to provide high quality EU information on population health, health determinants, and healthcare systems for innovative research and evidence-informed policy-making [7].

**Table 1:** Joint Action on Health Information Fact Sheets. Technical Dialogues meetings, 2019 and 2020 [8, 9]

Fact-Sheet	Topic
1	Prioritizing health information at national level
2	Capacity building activities under the European Health Examination Survey
3	Contributions to European Health Information Training Program (EHITP)
4	Health Information Training Course and roadmap for sustainability
5	Connecting health information system’s stakeholders through national nodes
6	Health data collection methods and procedures
7	Guidance for health reports
8	A sustainable ECHI shortlist
9	Innovative use of data sources
10	Use of artificial intelligence for public health surveillance
11	Burden of disease
12	Methodological guidelines to estimate health indicators using linked data and machine learning techniques
13	Non-health related EU databases for health surveillance. Case study industrial pollution and cancer
14	Composite health indicators for monitoring NCD: Hospital admissions and mortality ratio
15	Interoperability

The agenda and methodological approach for the meetings consisted of presentations by InfAct beneficiaries of the technical outcomes of InfAct extending the information provided in the Fact-Sheets [8, 9]; followed by a non-structured interaction, exchange of information, discussion and suggestions by the NTE.

The outcomes of the non-structured discussions were summarized, integrated and collected in Minutes of the TD. The NTE received a draft of the minutes one month after each meeting to provide additional comments and suggestions and to accept the final version by consensus.

### The Assembly of Members (AoM)

The AoM provided a platform between InfAct and national government representatives (one from the MoH and one from the MoR) to discuss the strategic vision for DIPoH and InfAct outcomes [7, 9] and to provide feedback, political guidance, and support to their future short and long term implementation. Additionally, the AoM has set the basis for a permanent governance body of DIPoH [9].

A total of 30 representatives of MoH and MoR from 22 EU/EEA MSs have participated actively in the meetings. The participating countries included: Austria, Belgium, Bosnia and Herzegovina, Czech Republic, Croatia, Estonia, Finland, France, Greece, Iceland, Ireland, Italy, Lithuania, Malta, Norway, Netherlands, Portugal, Spain, UK, Romania, Serbia, and Luxembourg.

Originally three meetings were scheduled to be held during the project, the first one to present InfAct to the representatives and to accept the Terms of Reference of the AoM and the other two meetings after the two TDs. However, it was necessary the organization of two additional meetings with the aim of discussing: the impact of COVID-19 pandemics in the project, the proposal for DIPoH [7], the European Strategy Forum on Research Infrastructures (ESFRI) application for the DIPoH and the setting up of PHIRI “as a practical rollout of DIPoH in the pandemic context” [9, 10]. Thus, a total of five AoM meetings were held. The first two meetings were face-to-face in March 2019 in Madrid and November 2019 in Brussels. The last three meetings (June 2020, October 2020, and May 2021) were held virtually due to the COVID-19 pandemic’s travel restrictions.

The Fact-Sheets [8, 9], the Minutes of the TD meetings, and more extended information of the DIPoH [7] were provided to the AoM representatives one month before each AoM meeting (Table 1).

**Fig. 1:**
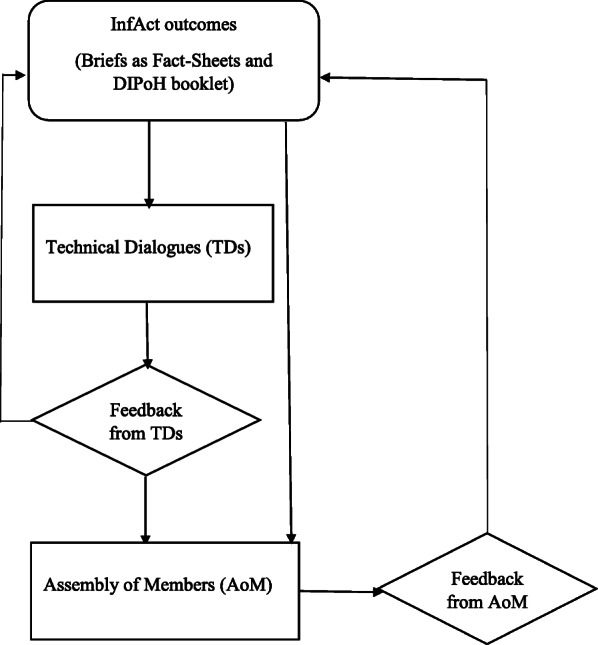
Information flow of InfAct outcomes to be discussed in the Technical Dialogues and the Assembly of Members and final integration of their feedback

During the AoM meetings, non-structured discussions were held based on: InfAct main outcomes presented by InfAct beneficiaries, Fact-Sheets and TDs Minutes with technical recommendations and suggestions (Fig. 1). All participating MSs representatives were able to provide their inputs related to the new proposed research infrastructure (DIPoH).

The outcomes of the non-structured discussions from the AoM representatives about the strategic vision for DIPoH including the feedback, advice, and requirements for their support were summarized, integrated and detailed in the Minutes of each AoM. Within one month of each meeting the AoM representatives had the chance of reviewing the minutes and to provide additional comments and suggestions. All participants accepted the final versions [9].

Additionally, structured letters of political support were provided to the AoM representatives, for them to consider providing their MS support for DIPoH to be added to the ESFRI 2021 roadmap.

## Results

### The Technical Dialogues

NTE participating in TDs were able to share clear and realistic needs, gaps in HI (in their own country and at EU level), and a wide range of views and concerns for the future. The NTE’s comments and suggestions were always submitted to the research and health authorities’ for them to make the final decision about whether to provide political support to InfAct’s outcomes including DIPoH; however, it is important to highlight that the NTE technical assessments were positive regarding the added value of InfAct’s outcomes.

Regarding InfAct’s main outcomes, the TD meetings reached the following consensus, that are detailed in the agreed minutes [9]:

- InfAct outcomes provided highly valuable tools and guidelines to be used nationally by experts.
- The proposed research infrastructure DIPoH is useful for technical mutual learning and cooperation among and within countries.
- NTE considered obtaining quality, accurate and robust health data as important and desirable goals.
- The feasibility to take up InfAct’s deliverables into HIS at national and EU level was considered complex. The main difficulties raised were: the different data quality and HI methods between countries, different functional and organizational approaches stablished in different countries, and different MSs’ interests.
- The capacity building activities provided by InfAct were considered valuable and needed at EU level.
- Additionally, the European General Data Protection Regulation (GDPR) was considered a possible technical concern in terms of HI interoperability between the countries in general. However, it was acknowledged that InfAct had made an assessment of GDPR interpretation and implementation in different countries and delivers possible options for the interoperability of health data in the EU respecting GDPR [11]. On the other hand, the NTE considered the anonymization of data an important concern. They suggested the elaboration of consensus guidelines about anonymization to be considered in DIPoH’s data management plan.

Overall, the technical outcomes presented by InfAct in the TD meetings were considered valuable for establishing priorities to advise decision makers.

The DIPoH proposal and its submission to the ESFRI roadmap were welcomed, but there were discussions about difficulties that may be faced. Country-specific organizations, financial issues and political positions were highlighted as challenging factors to implement DIPoH.

### The Assembly of Members

The AoM meetings reached the following consensus that are detailed in the agreed minutes [9]:

- All countries’ representatives agreed in the interest of setting up an integrated and sustainable HI infrastructure gathering population health information in order to support health research and evidence-informed policy-making.
- DIPoH was welcomed, and it was considered to be well-articulated and defined. Specifically, it was appreciated that several options for an EU-HIS had been investigated, providing their advantages and disadvantages [2]. It was also valued that the ESFRI roadmap application for DIPoH included different financing options as requested by the MSs. In summary, eleven countries and sixteen institutions supported the proposal, either by letters of political support or by signing the Memorandum of Understanding (MoU) and three countries, additionally, provided expression of financial commitment, for DIPoH to be added to the ESFRI 2021 roadmap.
- EU institutional interaction was highlighted as a key necessary value.
- The representatives valued the setting up of the Population Health Information Research Infrastructure (PHIRI) (funded by the Directorate-General for Research and Innovation Grant Number 101018317) [10] as a practical roll out of DIPoH for COVID-19 that will further implement DIPoH infrastructure and services.
- The representatives considered that the AoM, as a forum, was the most suitable way to inform all EU/EEA MSs on DIPoH’s progress and InfAct’s outcomes.

The main barriers for providing DIPoH political support were: future financial support, the commitment of the different budget providers among government departments (Research or Health), different strategic interests depending on departments, MSs internal and bureaucratic procedures, and the need for a clear benefit at national level. Finally, some MSs still had some concerns if a Research Infrastructure is the ideal format for an EU-HIS. They suggested that they would instead prefer an enlargement of the scope of ECDC to cover areas beyond infectious diseases, including chronic diseases and HSP. The need for wide country participation to maximize the added value of DIPoH was also stressed to increase country adherence to the initiative.

## Discussion

As a result of the discussions and the feedback provided by the participants from the TDs and the AoM boards, some important conclusions were drawn:

First, the early involvement of both the technical and political boards (in a multi-sectorial approach since the beginning of the project) had important benefits in guiding the development of DIPoH to support evidence-informed policy-making, because the boards provided the necessary feedback and suggestions about the requirements they would find appropriate for DIPoH’s future support, acceptance, and sustainability. This conclusion can be drawn from the members’ statement (expressed during the meetings and collected in the minutes) that their recommendations were needed to improve the proposal, in order to accept and support the long-term establishment of DIPoH in some countries. The proposal’s progressive evolution, including the members’ recommendations from each meeting, was appreciated and acknowledged by the representatives when the final proposal was presented in the last AoM meeting.

Second, to involve the NTEs and the political authorities in a multi-sectorial approach was beneficial to obtain governmental approval and commitment for the sustainability of DIPoH. Moreover, the approach of establishing a forum involving NTEs to provide their technical opinions to the political stakeholders to make evidence-informed political decisions is in line with the definition of evidence in health established by the European Advisory Committee on Health Research (EACHR) as “findings from research and other knowledge that may serve as a useful basis for decision-making in public health and health care” [12].

Furthermore, the involvement of both, decision-makers and technical boards, is in line with the WHO Regional Committee for Europe 66th session of September 2016 that declared:

“To improve the linkages between available evidence and policies, producers of knowledge (researchers) and users of knowledge (policy and decision-makers) need to have opportunities and a formal forum for exchange” [13]. Although in InfAct the TDs and the AoM meetings were arranged in different meetings, both boards had the opportunity to interact: indirectly through documentation that included summary meetings (Fig. 1) (InfAct Deliverables (https://www.inf-act.eu/assembly-members)) [9]; and by the direct exchange of information and recommendations at national level, especially through the set up of National Nodes by InfAct [14]. At TDs level, the analyses of the technical aspects of InfAct outcomes was essential to agree on the fact that the proposal was beneficial and necessary for each country. With this information, the political decision-makers participating in the AoM could focus on the necessary resources and the expected national returns to decide on its political commitment.

Third, the multi-sectorial approach included the involvement of political representatives both from the MoH and MoR; because a possible obstacle for the development of DIPoH was that in many countries, the research responsibility and funding is within the MoR while a Health related Research Infrastructure falls in the responsibility of MoH. By bringing together those representatives from the same country to the AoM, they were able to discuss their different perspectives and come together with a consensual decision about DIPoH. In the AoM meetings, it was also emphasized that high-level research (interest of MoR) would also support the needs and interests of MoH.

Fourth, with the involvement of technical and political national representatives, written commitment for the future sustainability of the infrastructure was obtained. At the present time, eleven countries provided letters of political support, three countries provided expression of financial commitment and sixteen institutions supported the project signing the Memorandum of Understanding, for DIPoH to be added to the ESFRI 2021 roadmap.

### Limitations

The implementation of the TDs and AoMs in InfAct has shown several limitations. First, the involvement and participation of high political representatives from 28 EU-MSs and 5 EEA countries was complex due to their agendas. However, participation of the 75% of the countries was achieved. Second, the country representative was not always the same person for all the AoM meetings. Third, due to COVID-19 travel restrictions, three out of five AoMs and one out of two TDs were held virtually. Finally, despite the positive assessment by TDs and the AoM representatives, most of InfAct’s outcomes were not always translated to a MS’s political commitment for DIPoH’s ESFRI roadmap application.

## Conclusion

Both boards described here, the TDs and the AoM, provided different but complementary and necessary inputs. The TDs focused mainly on added value for HI, feasibility aspects, and new technical and scientific adaptation required from current systems. At the same time, the AoM was more oriented towards resources, necessary country commitments, and national benefits. The outcomes of both boards provided the necessary advice and political support needed for DIPoH, and they are advisable for any EU population health purpose requiring MSs engagement.

## Data Availability

All data generated or analyzed during this study are available from InfAct and in the following links: www.inf-act.eu https://www.inf-act.eu/assembly-members

## Abbreviations

InfAct: Information for Action
HIS: Health Information Systems
NCD: Non-communicable Diseases
HSP: Health Systems Performance
EU-EEA: European Union European Economic Area
MSs: Member States
NTE: National Technical Experts
